# Influence of endocrine therapy on the ratio of androgen receptor (AR) to estrogen receptor (ER) positive circulating epithelial tumor cells (CETCs) in breast cancer

**DOI:** 10.1186/s12967-018-1724-z

**Published:** 2018-12-14

**Authors:** Monika Pizon, Daniel Lux, Ulrich Pachmann, Katharina Pachmann, Dorothea Schott

**Affiliations:** Transfusion Center Bayreuth, Bayreuth, Germany

**Keywords:** Breast cancer, Circulating epithelial tumor cells, Androgen receptor, Androgen to estrogen ratio

## Abstract

**Background:**

The androgen receptor (AR) is expressed in the majority of breast cancers and across the main breast cancer subtypes. Despite the high frequency of AR expression in breast cancer its appraisal remains controversial because its role is complex, dependent on the hormonal milieu. The aim of the current study was to investigate the frequency of AR and ER positive CETCs in breast cancer patients.

**Methods:**

The number of vital CETCs was determined from blood of 66 patients suffering from breast cancer and the expression of AR and ER on these cells was investigated using the maintrac method.

**Results:**

Numbers of CETCs/mL blood were significantly higher in patients with advanced disease as compared to patients with early stage disease. The fraction of AR positive CETCs was significantly higher than the fraction of ER positive CETCs (90% vs. 50%; P < 0.001). Patients with positive lymph nodes had less AR positive CETCs as compared to patients with negative lymph node status. The AR:ER ratio was higher in patients receiving tamoxifen therapy as compared to patients without tamoxifen therapy whereas treatment with aromatase inhibitor had no influence on AR:ER ratio.

**Conclusions:**

The ratio of AR to ER positive CETCs, obviously, is influenced by endocrine therapy, more specifically therapy with tamoxifen. Since AR expression seems to be one of the possible mechanism of resistance to endocrine therapy this may provide a new biomarker to select patients who might benefit from combination treatment of ER and AR inhibitors.

## Background

The biology of the androgen receptor (AR) and its therapeutic importance have been investigated extensively in prostate cancer [1]. However, AR is also widely expressed in other human tissues, including testis, ovary and breast [2]. In recent years AR has attracted a great deal of attention in the management of breast cancer, because it turned out that AR is expressed in about 80% of breast cancers depending on the subtype, often at a higher level than ER [2–4]. Like ER and PgR, AR is a nuclear transcription factor. The binding of steroid hormone androgens activates AR [5]. According to reports [1], over 70% of ER-positive breast cancers, approximately 60% of HER2-positive breast cancers, and 30% to 45% of TNBC (triple negative breast cancers) express AR. It has a multifunctional role and there is still a conundrum as to whether it acts as a tumor suppressor or an oncogene. Similar to ER and PR, AR expression is an important prognostic factor for disease free and overall survival [6, 7]. In addition, it is an independent prognostic marker associated with favorable clinico-pathological features, and a predictor of response to chemotherapeutic agents [1, 8]. AR expression is associated with a well-differentiated state and with more indolent breast cancers [6, 7]. In contrast, some studies showed that AR promotes the growth of ER+ breast cancer and TNBC via distinct mechanisms [9]. Furthermore, AR expression is associated with resistance to anti-estrogen therapies [1]. Resistance to established endocrine therapies is a well-documented phenomenon occurring de novo in 30% to 50% of all ER+ tumors and ultimately all metastatic ER+ breast cancers. It could result from tumor cell adaption from estrogen dependence to androgen dependence [6]. Recent studies suggest that not the expression level of AR alone predicts benefit from adjuvant endocrine therapy with tamoxifen but its relation to ER expression levels in primary tumors is of importance [5]. Thus, suboptimal response to ER-directed endocrine therapy may be due to the AR:ER ratio. A high AR:ER protein ratio has been reported to be indicative of a shorter time to relapse in patients treated with tamoxifen [6]. However, despite these emerging data, the role of AR in breast cancer is still not fully elucidated and the biology of AR in breast cancer remains incompletely understood [1]. No biomarkers are currently available to track changes in AR expression in blood over time in response to AR-targeting treatment. Circulating epithelial tumor cells (CETCs) used as “liquid biopsy” may become a tumor-specific biomarker of response to therapy. Circulating tumor cells are rare tumor cells that escape from solid tumors, travel into peripheral blood and can seed distant metastases. They have been reported to be a surrogate marker for tumor treatment response in primary breast cancer [10], and their presence has been linked to shorter survival in patients with metastatic breast, prostate, colorectal and lung cancer [1]. The purpose of our study was to better characterize AR and ER expression on CETCs in breast cancer contributing a new biomarker for targeted AR therapy, especially in patients with tamoxifen resistance.

## Methods

### Blood collection and processing

Peripheral blood (7.5 mL) from 66 patients with breast cancer in different stages of disease was drawn into normal blood count tubes with ethylenediaminetetraacetic acid (EDTA) as an anticoagulant and processed within 48 h of collection. The sampling of peripheral blood was carried-out 6–12 weeks after end of standard therapy (tumor resection, adjuvant chemotherapy, adjuvant radiotherapy). In patients with local or distant recurrence the blood was collected prior to treatment of recurrent disease. In parallel, control blood samples were collected from 15 healthy female and male donors aged 20–40 years. The study was conducted in accordance with Good Clinical Practice Guidelines and the declaration of Helsinki.

### Maintrac^®^

For CETC enumeration and further characterization the maintrac^®^ approach was used as reported previously [11]. Briefly, 1 mL blood was subjected to red blood cell lysis using 15 mL of erythrocyte lysis solution (Qiagen, Hilden, Germany) for 15 min in the cold, spun down at 700 g and re-diluted in 500 μL of PBS-EDTA. 5 µL of fluorescein-isothiocyanate (FITC)-conjugated anti-human epithelial cell adhesion molecule antibody (EpCAM) (clone HEA-125, Miltenyi Biotec GmbH, Germany) at a final concentration of up to 10^7^ cells/100 µL cell suspension were added and incubated for 15 min in cold. The corresponding isotype control for EpCAM (Mouse IgG1 K FITC, Miltenyi Biotec GmbH, Germany) was used at the same final concentration. The samples were subsequently diluted with 430 µL PBS-EDTA. A defined volume of the cell suspension and propidium iodide (PI) (Sigma-Aldrich, USA) was transferred to wells of ELISA plates (Greiner Bio-one, USA). Analysis of red and green fluorescence of the cells was performed using a Fluorescence Scanning Microscope, ScanR, (Olympus, Hamburg, Germany), enabling detection and relocation of cells for visual examination of EpCAM positive cells. For data analysis we used the ScanR Analysis software (Olympus, Hamburg, Germany). Vital CETCs were defined as EpCAM-positive cells, with intact morphology lacking in nuclear PI staining and, only these cells were counted. We used fluorospheres (Flow-Check 770, Beckman Coulter) for daily verification of optical components and detectors of the microscope, which are required to ensure the consistent analysis of samples.

### Cell lines

**Fig. 1:**
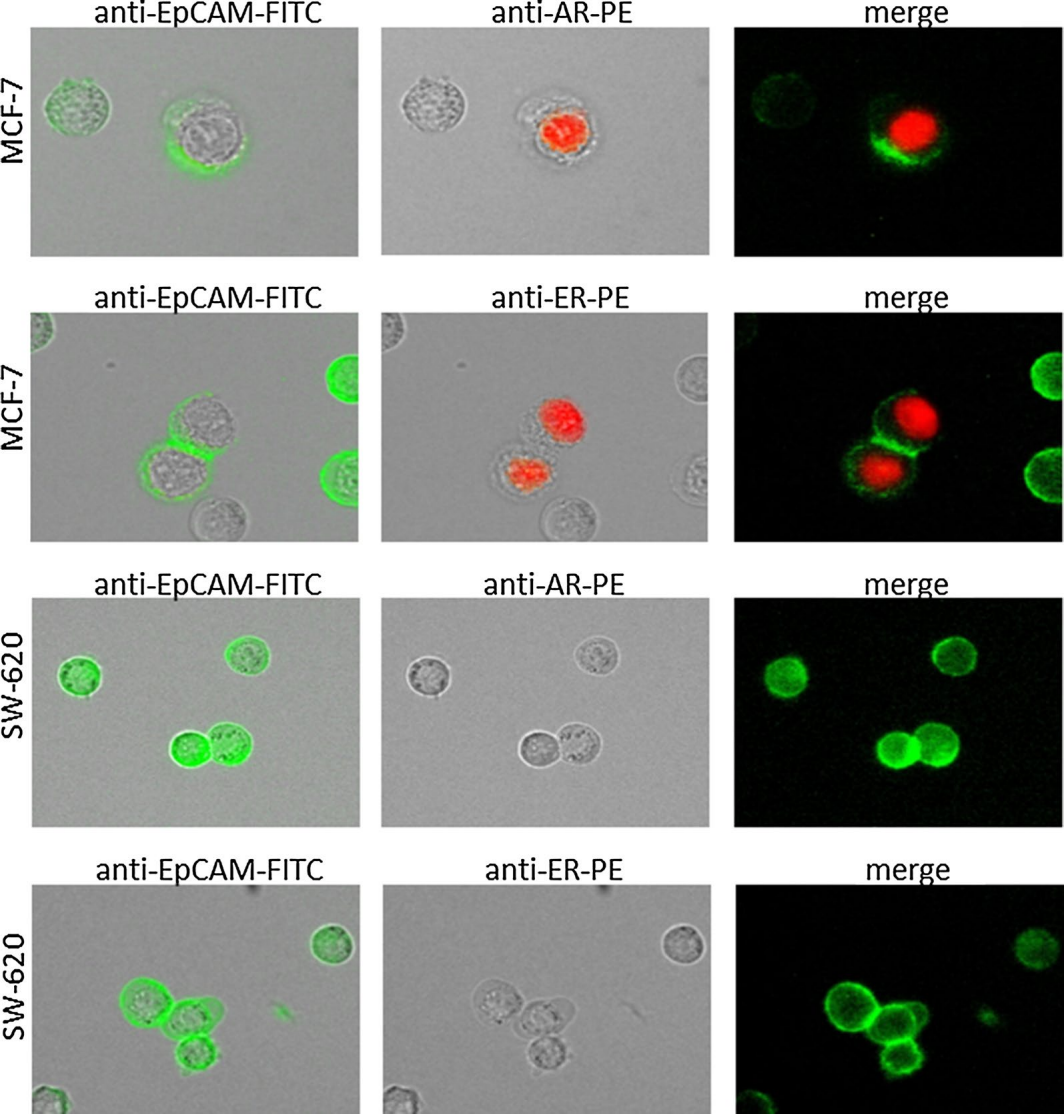
Positive and negative control for AR and ER staining. As positive control we use MCF-7 cell line. MCF-7 cells are positive for EpCAM (green) and AR (red) or ER (red). SW-620 cell line is used as a negative control. SW-620 cells are positive for EpCAM and strictly negative for AR or ER

For the AR staining Sk-Br-3 (data not shown) and MCF-7 cell lines were used as positive controls and the SW-620 cell line as a negative control (Fig. 1). For the ER staining MCF-7 cell line was used as a positive control and SW-620 cell line as a negative control. We obtained all cell lines from the CLS cell lines service (Eppenheim, Germany). MCF-7 cells were grown in Minimum Essential Medium Eagle ready-to-use medium (CLS cell lines service (Eppenheim, Germany). Sk-Br-3 and SW-620 cells were grown in Dulbecco’s modified Eagle’s medium with 4,5 g/L glucose, 2 mM l-glutamine (Gibco, Thermo Fisher Scientific, Waltham, USA) and 10% FBS. Cells were maintained at 37 °C in 5% CO_2_. For immunofluorescence analysis cells were detached from cell culture flasks using StemPro^®^ Accutase^®^ Cell Dissociation Reagent (Gibco, Thermo Fisher Scientific, Waltham, USA), washed and stained for AR or ER with the same protocol as patients’ samples.

### AR/ER-analysis

**Fig. 2:**
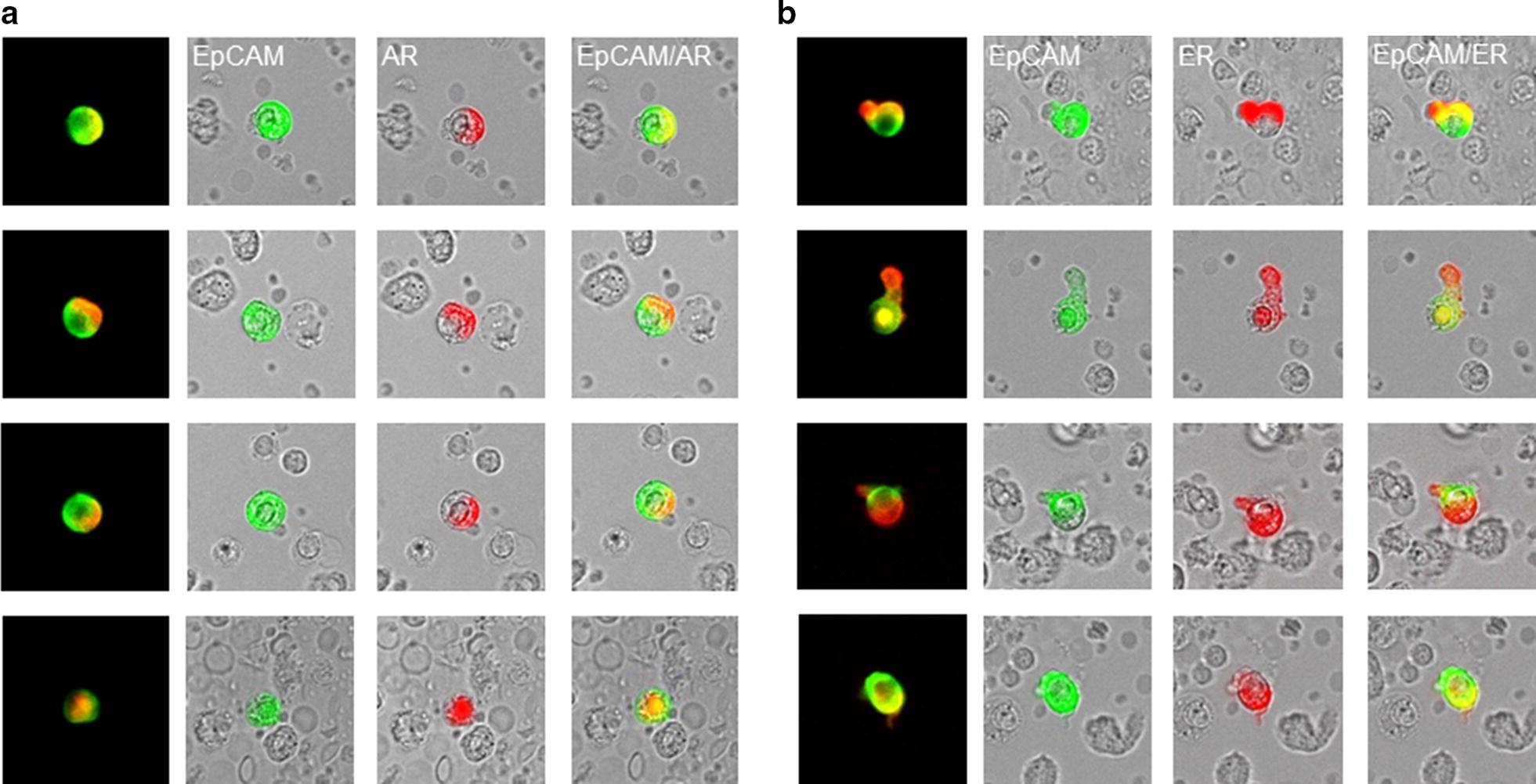
Androgen and estrogen expression on CETCs. **a** Representative images of EpCAM (green) and AR staining (red) on CETCs in one patient. **b** Representative images of EpCAM (green) and ER staining (red) on CETCs in the same patient. There is a substantial heterogeneity in EpCAM, AR and ER expression level across the CETCs from the same patient

The analyses of AR (Fig. 2a) and ER (Fig. 2b) expression on the CETCs were performed with an extended maintrac^®^ approach. For AR expression analysis we used an anti-human AR phycoerythrin (PE)-conjugated antibody (clone 523339, R&D Systems, Minneapolis, Canada) at a final concentration of 0.12 µg/mL and for ER we used an anti-human ER-PE conjugated antibody (clone E115, abcam, Cambridge, USA) at a final concentration of up to 10^7^ cells/100 µL cell suspension. The corresponding isotype controls for AR (Mouse IgG2B PE-conjugated Antibody, R&D Systems, Minneapolis, Canada) and ER (Rabbit IgG PE, abcam, Cambridge, USA) were used at the same final concentration. Finally, cells were visually inspected looking for a green and red surface staining, but also a well-preserved nucleus. For excluding expression of AR and ER on hematopoietic cells we additionally performed staining with EpCAM–FITC, AR–PE/ER–PE and CD45-Pacific blue antibodies (data not shown). The results for AR and ER were calculated as percentage of total number of CETCs.

### Statistical analysis

Statistical analysis was performed using the software programs SigmaPlot version 13.0 (Systat Software Inc., Chicago, USA) for Windows. Comparisons between the variables were performed with the Student t test (dichotomous variables) or ANOVA (variables with more than two categories), taking into account the possibility of using nonparametric tests. The correlation was calculated with the Pearson or Spearman rank correlation coefficient. Receiver operating characteristic (ROC) curve was used to find the optimal cut-off point of CETC counts. The optimal cut-off point was determined by choosing the CETC count with the highest sum of sensitivity and specificity regarding to the stage of disease. Area under curve (AUC) analysis was applied to demonstrate the performance for better distinguishing patients in early and advanced stages of disease. The significance level was P < 0.05.

## Results

**Fig. 3:**
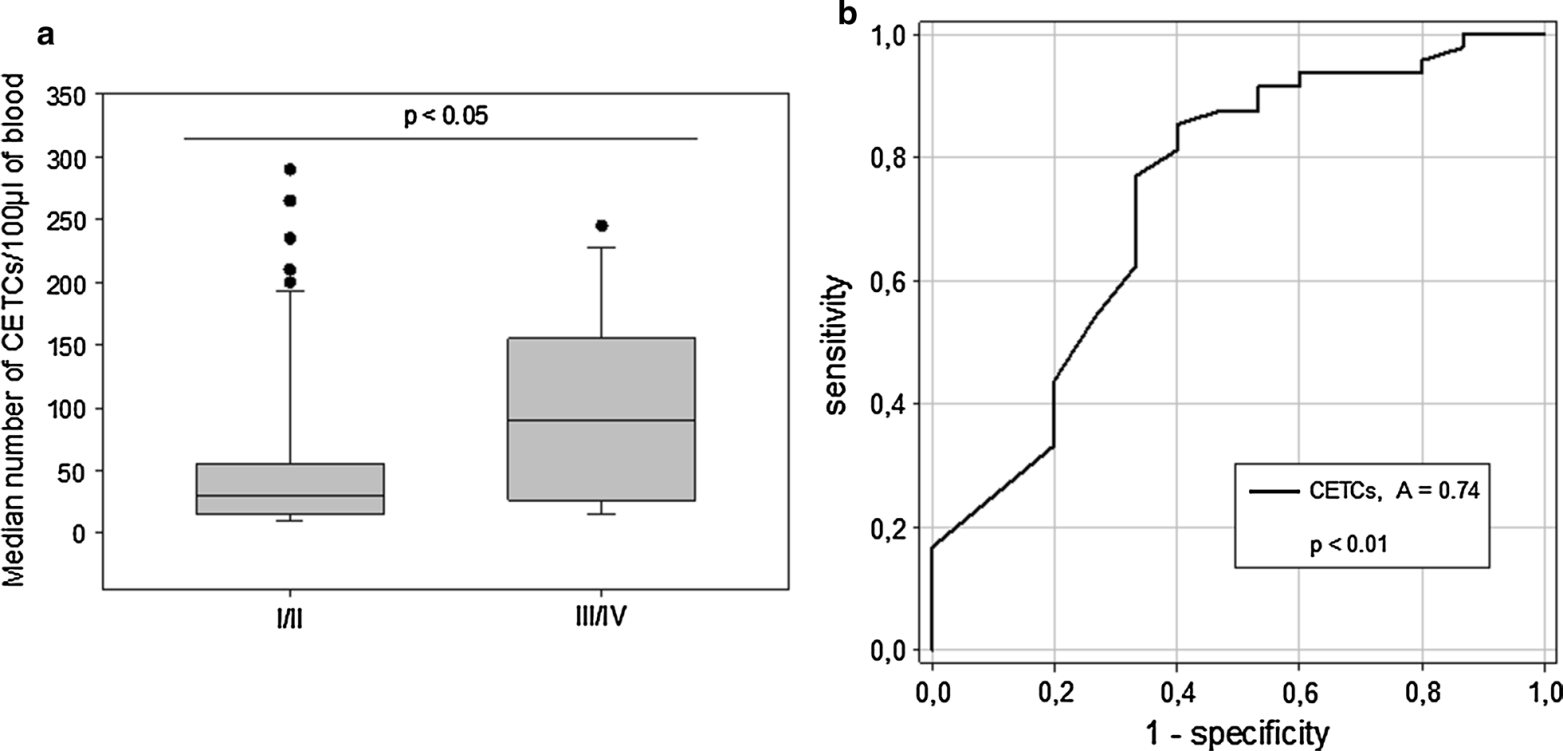
Number of CETCs in early (I/II) and advanced stage of disease (III**/**IV). **a** Box plot shows the number of CETCs in early and advanced stage of disease. Patients in advanced stage had significantly more CETCs as compared to patients in early stage of disease, *P < 0.05. **b** Determination of the cut-off point of CETC numbers by ROC curve analysis. ROC curve analysis was performed to determine the cut-off point of CETC numbers in terms of accuracy for discrimination of localized and advanced stage of disease. The value with the highest sum of sensitivity and specificity was chosen as a cut-off point: CETC number > 80 was determined as high CETC level and < 80 was determined as low CETC level. The AUC was 0.74, **P < 0.01

**Fig. 4:**
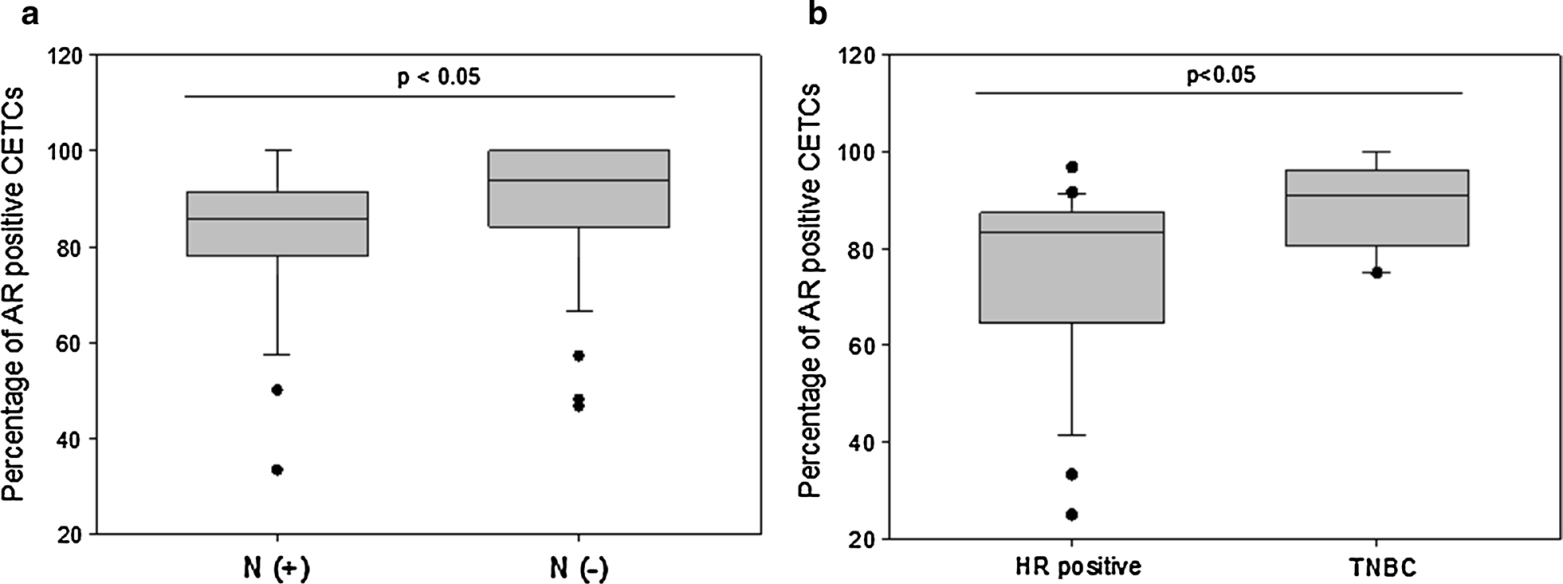
Box plot analysis of AR expression on CETCs in breast cancer patients with respect to lymph node and hormone receptor status. **a** We identified significant enhanced number of AR positive CETCs in patients without lymph node metastasis (N−) as compared to patients with lymph node involvement (N+), *P < 0.05. **b** In TNBC patients we observed elevated numbers of AR positive CETCs as compared to patients with HR positive primary tumors, *P < 0.05

**Fig. 5:**
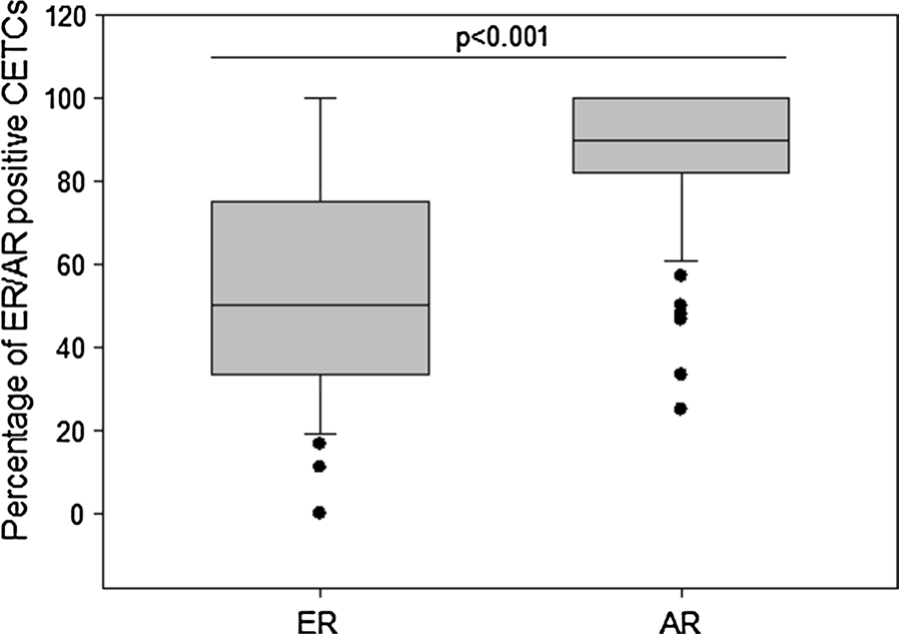
Box plot of ER and AR positive CETCs detected in breast cancer patients. The box plot shows higher AR expression on CETCs compared to ER expression on CETCs, ***P < 0.001

**Fig. 6:**
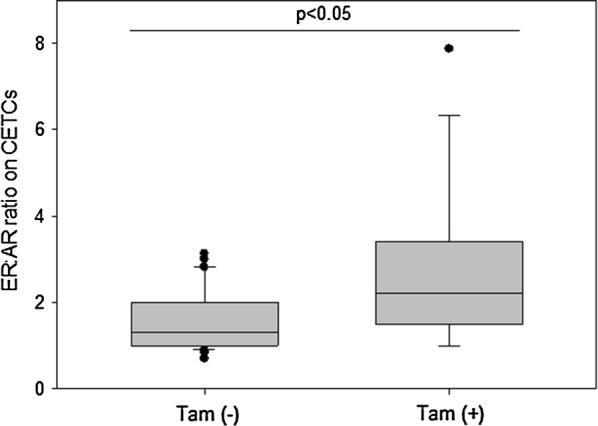
Box plot analysis of ER:AR ratio on CETCs by patients with and without tamoxifen therapy. In patients who received tamoxifen therapy we identified a significant enhanced ER:AR ratio as compared to patients without tamoxifen therapy, *P < 0.05

The patient characteristics according to AR and ER expression are shown in Table 1. 21 patients were classified as stage I (32%), 28 in stage II (42%), 9 in stage III (14%) and 7 in stage IV (11%). Patients with advanced stage of disease (stage III/IV) had statistically significantly more CETCs as compared to patients with early stages (stage I/II) of disease (P < 0.05) (Fig. 3a). The median number of CETCs in early stage of disease was 30 CETCs/100 µL of blood (ranging from 10 to 290), and in advanced stage of disease the median number of CETCs was 95 CETCs/100 µL of blood (ranging from 15 to 565). As negative control we tested blood samples from 15 healthy controls and confirmed that none of the samples were positive for CETCs. The receiver operating characteristic (ROC) curve shows that the number of CETCs was a predictor of advanced stage of disease. The AUC for number of CETCs was 0.74 (P = 0.01) (Fig. 3b). The cut-off number of CETCs predictive for advanced stage of disease for breast cancer was 80/100 µL blood with a sensitivity of 85% and a specificity of 60%. AR positive CETCs were observed in all examined patients. Median percentage of AR positive CETCs was 90% and ranged from 25 to 100%. The percentage of AR positive CETCs correlated with lymph node and hormone receptor status. Patients with a negative lymph node status had a statistically significantly higher fraction of AR positive CETCs as compared to patients with lymph node involvement (93% vs 86%, P < 0.05) (Fig. 4a). In addition, the percentage of AR positive CETCs was significantly higher in TNBC as compared to hormone receptor positive patients (median 91 vs 83, P < 0.05) (Fig. 4b). ER positive CETCs could be observed in 94% of patients. Median percentage of ER positive CETCs was 50% and ranged from 11 to 100%. Interestingly, the fraction of AR positive CETCs was significantly higher than the fraction of ER positive CETCs (90% vs. 50%; P < 0.001) (Fig. 5). Women who received tamoxifen therapy had a higher ratio of AR-positive:ER-positive cells as compared to women not treated with tamoxifen (2.2 vs 1.3, P < 0.05) (Fig. 6) whereas there was no such association between the AR:ER ratio and treatment with aromatase inhibitors (AIs).

**Table 1:** Patient characteristics and CETC examination results

Clinico-pathological characteristics	Number of patients (%) with CETCs	Median of CETC counts	*P*-value	Median (%) of AR positive CETCs	*P*-value	Median (%) of ER positive CETCs	*P*-value
Age (years)							
≤ 50	26 (39)	85	*0.001*	90	0.98	50	0.91
> 50	40 (61)	30		89		50	
Tumor size							
T1	29 (44)	35	0.34	83	0.42	50	0.86
T2	34 (51)	35		80		50	
T3	2 (3)	120		73		62	
n.a	1 (2)						
Lymph node metastasis							
Positive	25 (38)	40	0.63	86	*0.04*	50	0.63
Negative	40 (61)	35		93		50	
n.a	1 (1)						
Distant metastasis							
Positive	7 (11)	85	0.11	88	0.99	49	0.97
Negative	58 (88)	35		89		50	
n.a	1 (1)						
Stage							
I/II	49 (74)	30	*0.009*	90	0.93	51	0.16
III/IV	17 (26)	95		89		62	
HER2 status							
Positive	21 (32)	35	0.58	89	0.93	56	0.37
Negative	45 (68)	35		90		52	
ER/PR status							
Positive	51 (77)	35	0.97	87	0.74	52	0.82
Negative	15 (23)	35		88		46	
Radiation							
Yes	24 (36)	40	0.93	92	0.19	48	0.27
No	34 (52)	40		88		52	
n.a	8 (12)						
Hormone receptor status							
TNBC	10 (16)	55	0.31	91	*0.02*	56	0.55
ER/PR pos.	51 (84)	35		83		46	
Hormone therapy							
Yes	33 (50)	35	0.77	89	0.57	47	0.07
No	32 (49)	35		91		60	
n.a	1 (1)						
Chemotherapy							
Yes	30 (45)	50	0.51	90	0.47	52	0.96
No	36 (55)	35		88		48	
Tamoxifen therapy							
Yes	22 (33)	35	0.64	90	0.75	45	*0.042*
No	43 (66)	35		91		58	
n.a	1 (1)						
Aromatase inhibitor							
Yes	19 (29)	50	0.29	85	0.22	50	0.35
No	46 (70)	35		91		39	
n.a	1 (1)						

Statistically significant values are in italics

## Discussion

The detection of tumor cells circulating in the peripheral blood of metastatic cancer patients has been associated with both metastatic disease and a higher risk of progression [12]. With the maintrac^®^ method, an approach designed to minimize cell loss during the labeling and analysis process we were one of the first who could detect circulating tumor cells also in non-metastatic patients. Cell numbers could be monitored during treatment and we could show that an increase in the number of CETCs during chemotherapy correlates with an unfavorable prognosis [10]. Similarly, an association between a poor prognosis and persisting numbers of circulating tumor cells following chemotherapy in early stage of disease has been reported by Rack et al. [13]. Using the maintrac^®^ approach the number of CETCs from the peripheral blood of patients with solid tumors detected is higher than that detected with other methods in both, early and advanced stage of disease [14]. This is due to the omission of all enrichment steps during the sample preparation. In opposition, the fixation and magnetic enrichment in the CellSearch^®^ method leads to a massive destruction of the tumor cells and also to a heavy cell loss [14]. However, preservative agent present in the CellSave tubes provide poor antigen preservation due to the cross-linking mechanism of fixation [15].

In primary disease the number of CETCs was significantly related to the stage of disease. Our results indicate that patients with higher CETC counts have a more advanced stage of disease than patients with lower CETC counts. The AUC to distinguish between early and advanced stage with the CETC count was 0.74 with a high sensitivity and specificity. Thus, the CETC count in the peripheral blood is predictive for the severity of cancer disease. Our results are in agreement with the results of Maestro et al. [16] who used the numbers of circulating tumor cell to determine the cut-off value for discrimination between localized and metastatic stages with an AUC for breast cancer cases of 0.76. This indicates that the number of CETCs can be used as an additional predictive marker for better distinguishing patients in early and advanced stages of disease. AR is expressed in about 70–90% of breast cancers and seems to play a major role in carcinogenesis. Currently, it is under investigation in the clinical setting as a therapeutic target [17]. In contrast to Fujii et al. [1] who found only in 18% of metastatic breast cancer patients circulating tumor cells positive for AR we were able to detect AR positive CETCs in all patients. Reyes et al. [18] observed AR positive circulating tumor cells in 100% of metastatic castration-resistant prostate cancers. Data regarding clinicopathologic parameters of tumor in relation to AR status are inconsistent. Some studies report the observation that there is no correlation between AR expression and lymph node involvement and other conventional parameters [19, 20]. Ogawa et al. [21] in contrast reported that AR expression is a favorable biomarker related to lymph node metastasis. Patients with negative lymph nodes had a higher rate of AR positive tumors. Our results show that a high rate of CETCs with AR expression is a sign of more well-differentiated state with a decreased aggressiveness. Patients without lymph node involvement had a higher percentage of AR positive CETCs as compared to patients with lymph node involvement.

Definition of triple negative breast cancers (TNBC) is the absence of expression for ER and PR and absence of overexpression for HER2 by immunohistochemistry (IHC). It is well known that TNBC patients have more frequently axillary lymph node involvement and a shorter overall survival. Due to the lack of a receptor target chemo- and radiotherapy represent the only option for the treatment of TNBC [22]. In the literature the frequency of AR expression in TNBC is very variable ranging from 7 to 75% [22]. The prognostic value of AR expression in TNBC is not well understood and still controversial. On the one hand studies showed that patients with positive AR expression have poor overall survival, however, on the other hand other studies documented the opposite conclusion [23]. To the best of our knowledge we are the first who report about expression of AR on CETCs in TNBC. We found that TNBC patients had a higher percentage of AR positive CETCs as compared to patients with a hormone receptor positive primary tumor. Therefore, AR targeted therapy might become a new option in TNBC therapy. Furthermore, monitoring AR positive CETCs during androgen blockade could reflect the efficacy of therapy.

Our results in hormone receptor positive breast cancer are in agreement with the results from xenografts from cell lines reported by D’Amato et al. [24]. Statistically significantly more AR positive CETCs were detected in TNBC as compared to ER positive CETCs. Thus, AR overall is more frequently expressed in CETCs than ER or PR, however, the role of AR is complex, dependent on the hormonal milieu and remains controversial [24]. There are three types of resistance to SERM therapy as described by Fan and Jordan: metabolic resistance, de novo resistance and acquired resistance [25, 26]. More recently it has been demonstrated that another mechanism of resistance to anti-ER therapies may be the adaptation of the tumor from estrogen to androgen dependence [7, 24]. In hormone receptor positive breast cancer AR is frequently expressed in tumor tissue and related to prognosis. It is expected to be used as a predictive marker for hormone therapy and a potential therapeutic target for breast cancer [7]. Resistant cell populations seem to be in permanent evolution depending on selection pressure that enhances survival of new clones [27], this is corroborated by our results showing that patients with tamoxifen therapy had a higher AR:ER ratio as compared to patients without endocrine therapy whereas treatment with aromatase inhibitor had no influence on AR:ER ratio. In vitro studies have demonstrated that tamoxifen-therapy resistant tumors express higher level of AR and lower ER levels than tamoxifen-therapy sensitive tumors [28, 29]. Rangel et al. [30] reported a high AR:ER ratio to be associated with aggressive biological features and worse prognosis. Furthermore, Cochrane and coworkers demonstrated that AR:ER ≥ 2 was associated with an increased risk of tamoxifen failure in breast cancer [6]. If tumors with a high ratio of AR:ER expressing circulating tumor cells turn out to be resistant to tamoxifen therapy and this raises the question whether a blockade of AR may be an effective and new target to overcome tamoxifen resistance. Our study has some limitations. The study population is relative small and follow-up data for disease free and overall survival are missing. To address these limitations future studies need to include a larger cohort of patients and follow-up of patients.

## Conclusion

In conclusion, with the maintrac platform we are able to identify CETCs in patients with breast cancer and to determine AR expression on those cells. Results from our study suggest that AR expression detected on CETC has capability to be a biomarker for identifying patients who might develop tamoxifen resistance. This needs to be clinically validated.
